# Triage Nurses’ Perceptions of Patient Safety Errors During Triage in the Emergency Department: Qualitative Thematic Analysis Study

**DOI:** 10.3390/healthcare14152291

**Published:** 2026-07-29

**Authors:** Zvonka Fekonja, Urška Fekonja, Maja Gradišnik

**Affiliations:** 1 Faculty of Health Sciences, University of Maribor, 2000 Maribor, Slovenia; 2 Emergency Department, University Clinical Centre Maribor, 2000 Maribor, Slovenia; ufekonja@gmail.com (U.F.); majjagradisnik@gmail.com (M.G.)

**Keywords:** patient safety, triage, emergency service, qualitative research, patient care

## Abstract

**Background/Objectives**: Patient safety is a key component of emergency care, and triage is essential for timely patient assessment and prioritisation. Errors during triage may compromise patient outcomes and quality of care. While previous studies have focused on triage accuracy, little is known about triage nurses’ perceptions of patient safety errors. This study aimed to explore triage nurses’ perceptions of patient safety errors during triage encounters. **Methods**: A descriptive qualitative study was conducted in the emergency department of a large university hospital in Slovenia. Nineteen triage nurses participated in semi-structured interviews between September and December 2024. Interviews were audio-recorded, transcribed verbatim, and analysed using inductive thematic analysis. The study followed the Standards for Reporting Qualitative Research (SRQR). **Results**: One overarching theme, Perceptions of patient safety errors during triage encounter, and five sub-themes were identified: Types of errors; Causes of errors; Consequences of errors; Accountability culture; and Error prevention. Participants described administrative errors, including patient misidentification and inaccurate data entry, and procedural errors, such as incorrect triage categorisation and under- or overtriage. Errors were linked to human and organisational factors and high patient volume. Consequences included delays in care and potential patient harm. Prevention strategies included education, adequate staffing, communication, teamwork, and systematic patient identification. **Conclusions**: Patient safety errors in triage are perceived as multifactorial events influenced by individual, organisational, and system-level factors. Continuous training, adequate staffing, effective communication, and a supportive, non-punitive safety culture may contribute to reducing errors and improving the quality and safety of triage practice.

## 1. Introduction

Ensuring patient safety is fundamental to providing high-quality care and reducing potential harm during health care delivery [1]. Among the critical points of patient interaction in the emergency department, triage is a crucial stage that requires rapid and accurate assessment of a patient’s health status [2] to identify those at risk of serious clinical conditions, who require urgent or specialized care [3]. Effective triage relies on both a validated triage system and the clinical judgment, experience, and intuition of the triage nurse [4]. Over the past decades, several standardized triage systems have been developed worldwide to prioritise healthcare needs according to their severity [2,5]. Triage scales assist the triage nurse in quickly assessing and prioritising care based on the patient’s condition [6]. Despite the recognised importance of triage for ensuring timely and safe patient care [2], the concept of patient safety errors in this context remains heterogeneous in the literature. Clarifying what constitutes a patient safety error is, therefore, essential for interpreting the findings of this study.

In the context of this study, patient safety errors are unintended failures in the triage process that could compromise patient safety, regardless of whether patient harm occurs [7]. These errors may include administrative errors (e.g., patient identification or documentation errors) and procedural errors (e.g., inappropriate triage categorisation or clinical assessment errors) [2,8,9]. Patient safety errors should be distinguished from adverse events, which result in patient harm, and near misses, which are patient safety incidents that do not reach the patient or do not result in harm because they are intercepted or occur by chance [7]. This conceptual distinction provided the framework for interpreting triage nurses’ perceptions of patient safety errors explored in the present study.

Within the context of triage, patient safety errors are commonly discussed in relation to undertriage and overtriage [4,10]. Therefore, according to Ausserhofer, Zaboli, Pfeifer, Solazzo, Magnarelli, Marsoner, Siller and Turcato [2], triage accuracy is crucial, as neither undertriage (i.e., underestimation of acuity or severity of the condition) nor overtriage (i.e., overestimation of the acuity or severity of the condition) is considered appropriate triage outcomes. In triage systems, the characteristics of the Registered Nurse are critical to accurately assessing the patient’s treatment priority and limiting errors [11], as both undertriage and overtriage can lead to poor patient outcomes. Other factors contributing to triage errors, according to Ausserhofer et al. [2], can be broadly categorized into patient-related and work environment-related factors. Most errors are not solely the result of individual performance but arise from systemic issues. These include poor work organization, lack of protocols and standards, inadequate resources, unfavourable working conditions, and communication failures [12]. Together, these factors reflect the broader organisational environment in which triage is performed and highlight the importance of fostering a strong patient safety culture. Within this context, accountability culture, closely aligned with the principles of a just culture, refers to an organisational approach in which healthcare professionals are encouraged to take responsibility for safe practice while recognising that many errors arise within complex healthcare systems [13]. Such a culture promotes transparency, learning from errors and continuous quality improvement rather than focusing primarily on individual blame [14]. In contrast, repeated exposure to demanding working conditions may contribute to the normalisation of errors, a process in which healthcare professionals come to perceive unsafe practices or errors as an expected part of routine clinical work, potentially reducing awareness of patient safety risks [15]. These concepts reinforce the view that patient safety errors should not be understood solely as individual failures but as outcomes of complex interactions within healthcare systems [16]. This systems perspective is further supported by Azarmi et al. [17], who describe systemic errors that can result from poor organisation of work processes, the absence of rules, norms, and standards, inadequate physical environments, lack of resources, and lack of communication.

Most published studies on triage have evaluated objective aspects of triage performance, such as triage accuracy, rates of undertriage and overtriage, agreement with reference standards, and factors associated with triage errors [2,4,10,11]. In contrast, relatively little attention has been paid to how triage nurses themselves perceive, interpret, and make sense of patient safety errors encountered in everyday clinical practice. Exploring these perceptions is important because healthcare professionals’ experiences provide insight into the organisational and contextual mechanisms that may contribute to patient safety errors, but cannot be fully captured through quantitative measures of triage accuracy alone.

Although previous studies have generated important evidence regarding the accuracy and determinants of triage decisions, little is known about how triage nurses themselves perceive and interpret patient safety errors encountered during routine triage practice. Existing literature has primarily focused on the accuracy of triage decisions, while limited attention has been given to triage nurses’ perceptions of errors, representing a significant knowledge gap. Understanding nurses’ perceptions is essential for improving triage processes and enhancing patient safety. Therefore, this study aims to explore triage nurses’ perceptions of patient safety errors during the triage process. The research question guiding this study is: How do triage nurses perceive patient safety errors during the triage encounter?

## 2. Materials and Methods

### 2.1. Study Design

We used a descriptive qualitative design. The study was grounded in a constructivist paradigm, as the aim was to understand how triage nurses perceive and make sense of patient safety errors within their specific clinical context [18]. A constructivist paradigm was considered appropriate because it recognises that knowledge is socially constructed through individuals’ experiences and interactions. This perspective informed the interview process by encouraging participants to describe their own experiences and perspectives openly. It also guided the data analysis by focusing on the explicit meanings participants attributed to patient safety errors and informed the interpretation of the findings by recognising that these experiences were shaped by individual, organisational, and contextual influences rather than representing a single objective reality.

### 2.2. Study Sample and Setting

To explore triage nurses’ experiences of patient safety errors during triage, we conducted semi-structured interviews with 19 triage nurses working in the emergency department. Purposive sampling was used to identify potential participants who would provide rich and relevant insights on patient safety errors in the triage process. Eligible triage nurses who met the inclusion criteria were informed about the study by the first author during staff meetings and through individual contact in the emergency department. They received verbal and written information about the study and were invited to participate voluntarily. All eligible triage nurses employed in the emergency department who met the inclusion criteria were invited to participate in the study. Nurses who agreed to participate were invited to schedule an interview at a time convenient for them. All participants were triage nurses employed in one of the largest university hospitals in Slovenia. In Slovenia, the Manchester Triage System (MTS) has been selected as the triage model, based on national professional recommendations, as the most appropriate for the local context [19]. Furthermore, triage is conducted by a Registered Nurse who possesses advanced expertise in this field and has a minimum of three years of experience working in an emergency department. The inclusion criteria were as follows: being licensed as a registered nurse, having at least one year of emergency care experience, having completed a triage course, having direct experience in triage, and willingness to participate. Both male and female nurses were included, aged 24 to 56 years, with triage experience ranging from 1 to 6 years. Of the 19 nurses, 15 (78.95%) were female, and four (21.05%) were male. The mean age was 33.79 years (SD = 9.02).

### 2.3. Data Collection

The interviews were semi-structured and followed by an interview guide developed by the research team, based on relevant literature and the study aim. The interviews were conducted by the first author, who has over eight years of clinical experience in the emergency department. The interviewer’s clinical experience facilitated rapport with participants and deepened understanding of the emergency department context. At the same time, the researchers acknowledged that prior professional knowledge and assumptions could influence data collection and interpretation. Throughout the study, the interviewer maintained ongoing reflexive awareness by critically considering how prior clinical experience, professional assumptions, and interactions with participants could influence both the interview process and the interpretation of the data. To enhance reflexivity, the interviewer used open-ended questions, encouraged participants to elaborate on their experiences, and avoided leading questions. Throughout the interviews, attention was paid to allowing participants’ perspectives to emerge rather than confirming pre-existing assumptions. The interviewer was professionally familiar with the emergency department environment and was known to some participants through routine clinical practice. However, no direct supervisory, managerial, or evaluative relationship existed between the interviewer and the participants. The researchers acknowledged that this professional familiarity could have influenced participant recruitment and willingness to participate. To minimise this potential influence, all eligible nurses were approached using the same recruitment procedure, were assured that participation was entirely voluntary, and were informed that their decision to participate or decline would have no consequences for their professional role or employment. The interviewer acted solely as a researcher, and participants were encouraged to share their experiences openly and honestly. Each interview, which was limited to the interviewer and participant, began with a brief introduction to the study, obtaining informed consent, and permission to audio-record the interview. The interviews were based on a guide that focused on the triage process and experiences with patient safety errors during triage. The interview began by asking participants to describe the patient triage process in the emergency department. This approach enabled participants to define further their roles, prerequisites, and requirements for performing safe patient triage. Subsequent questions aimed to clarify the concept of errors and their impact on patient safety. The data collection guide included the following questions: (a) How would you describe a triage error? (b) Do you think there are errors in triage, and if so, what are they? Could you please describe them? (c) How do you think you would prevent these errors from occurring?

The interviews were conducted between September and December 2024 at a time convenient for the participants. After identifying eligible and willing participants, individual interviews were arranged with providers, and informed consent was obtained. All interviews took place at the participants’ workplace after working hours. Interviews lasted 10 to 43 min (mean duration = 17 min, SD = 8.44), and each participant was interviewed once. During the interviews, participants were encouraged to speak freely about their experiences of patient safety errors in the triage process. Interviews were recorded with informed consent and anonymised and later transcribed verbatim; linguistic omissions, repeated words, missing syllables, and single pronouns were omitted. No predefined sample size was established a priori.

Instead, interviews were conducted until data saturation was achieved, indicating that successive interviews yielded no new codes, categories, or insights relevant to the research question. Data saturation was assessed concurrently with data collection and preliminary data analysis. In this study, saturation was primarily understood as code saturation, defined as the point at which no new codes or concepts relevant to the research question emerged during the ongoing coding process. Data saturation was reached after 15 interviews. To confirm that no additional thematic variation would emerge (thematic saturation), four more interviews were conducted. These additional interviews did not generate any new codes, themes, or meaningful variations in participants’ accounts, confirming that sufficient depth and breadth of the data had been achieved. Therefore, all 19 interviews were included in the final analysis. The interviewer’s clinical expertise supported attentive listening and contextual understanding. Transcripts were not returned to participants for member checking because participants were recruited from a busy emergency department with rotating shift schedules, making repeated contact after the interviews difficult and potentially burdensome. Instead, credibility was enhanced through investigator triangulation, independent coding of a subset of transcripts by multiple researchers, iterative discussions to achieve consensus on codes and themes, and the use of illustrative participant quotations to ensure that the findings remained grounded in the original data. Each interview was labelled as an “TN1-TN19”. We adhered to the SRQR reporting standards for qualitative studies [20].

### 2.4. Data Analysis

Data were analysed using an inductive thematic analysis framework [21]. The analysis was conducted iteratively across six phases, with researchers moving back and forth between them throughout the analytical process. The analysis followed the six phases proposed by Braun and Clarke: familiarisation with the data; generating initial codes; searching for themes; reviewing themes; defining and naming themes; and producing the report. The coding process began with repeated reading of each transcript to achieve immersion in the data. Meaning units relevant to the research question were identified and assigned descriptive initial codes that remained close to participants’ own words. Coding was conducted at the semantic level, focusing on the explicit meaning of participants’ accounts rather than focusing on the interpretation of underlying latent meanings. Codes with conceptual similarities were then grouped into preliminary subthemes, which were continuously refined through constant comparison across interviews. This iterative process resulted in the development of an inductive coding framework that evolved throughout the analytical process. Rather than applying a predefined codebook, codes were generated, reviewed, refined, and organised into subthemes and overarching themes through regular discussion within the research team. Preliminary subthemes were subsequently reviewed, refined and integrated into overarching themes. The researchers moved repeatedly between the original transcripts, initial codes, subthemes, and themes, reviewing internal coherence within each theme and clear distinctions between themes until the final thematic structure accurately reflected participants’ experiences. Data were primarily coded and categorised by one researcher (ZF), and a subset of transcripts was independently coded by two researchers (UF & MG). To minimise researcher bias during the analytical process, emerging codes and themes were continuously compared with the original transcripts to ensure that interpretations remained grounded in participants’ accounts rather than researchers’ preconceptions. Emerging codes, subthemes, and themes were reviewed collaboratively by the research team, and any differences in coding or interpretation were resolved through iterative discussion and consensus. Reflexive discussions among the researchers encouraged critical examination of assumptions throughout the analytical process. Analytical decisions, coding revisions, and theme development were systematically documented, thereby maintaining an audit trail that enabled the research team to trace the development of codes and themes from the original data to the final thematic structure. To enhance methodological transparency further, a coding tree showing the development of themes (Supplementary Figure S1) and examples of the analytical process from meaning units to themes (Supplementary Table S1) are provided as Supplementary Material. Participant quotations are presented in the Results section to support transparency and credibility. All researchers used MAXQDA Analytics Pro version 12 software (VERBI Software GmbH, Berlin, Germany, 2018) for data management.

### 2.5. Rigour

Rigour was ensured by applying the trustworthiness criteria for qualitative research, as outlined by Lincoln and Guba [22]. Dependability was enhanced through systematic documentation of the research process, maintenance of an audit trail, and the use of an emergent design in which data collection and analysis were conducted concurrently. This iterative approach allowed the ongoing refinement of coding and interpretation as the analysis progressed. Interviews continued until data saturation was achieved, and the study context was described in sufficient detail to support the assessment of transferability. Detailed participant characteristics and the study setting are reported to enable readers to judge the applicability of the findings to other contexts. Credibility was strengthened through investigator triangulation, prolonged engagement with the data, repeated comparison of emerging codes and themes with the original transcripts, and iterative refinement of themes throughout the analytical process. While the first author conducted the primary coding, a subset of transcripts was independently analysed by two additional researchers, and coding decisions, emerging interpretations, and theme development were discussed until consensus was reached. Reflexivity was maintained throughout the analytical process by regularly questioning assumptions, discussing alternative interpretations, and comparing coding decisions to minimise the influence of individual perspectives, thereby enhancing confirmability. An audit trail documenting coding decisions, theme refinement, and analytical discussions was maintained throughout the study to support confirmability and dependability. The interviewer reviewed each transcript against the audio recording to ensure transcription accuracy. Although participant validation (member checking) was not undertaken, credibility was strengthened through collaborative analysis, consensus discussions, and the presentation of illustrative participant quotations, ensuring the findings remained grounded in the original data. The findings are presented with illustrative quotations to enhance their transferability [22]. Taken together, these strategies enhanced credibility, dependability, confirmability, and transferability of the study findings.

### 2.6. Ethical Considerations

The study design, data collection, and consent forms were approved by the National Medical Ethics Committee (Ref. No.: 0120-558/2017/14). Before commencing the study, participants were informed about the study, including its aims and methodology, were provided with written informed consent forms, and were reassured that the information they provided would be kept strictly confidential. Furthermore, they were informed that they had the right to withdraw from the study at any time. The confidentiality and anonymity of the data were ensured by removing identifiable information before data processing.

## 3. Results

A main theme, “Perceptions of patient safety errors during triage encounter”, and five primary thematic sub-themes were generated (Table 1):

- Types of errors;
- Causes of errors;
- Consequences of errors;
- Accountability culture;
- Error prevention.

Although presented as distinct themes, participants described these aspects of triage practice as closely interconnected. Different types of errors were perceived to arise from interacting human, organisational, and contextual factors, leading to consequences for patient safety, while an accountability culture influenced how errors were recognised and discussed. Strategies for error prevention were viewed as responses to these interconnected challenges rather than isolated interventions.

### 3.1. Types of Errors

The secondary level sub-theme, Types of errors, during the triage process consists of two primary level sub-themes, namely: (1) Administrative errors and (2) Procedural errors.

According to the interviewees, administrative errors often include errors in recording the patient identity, such as cases where a patient is incorrectly registered without proper identification:

*“… an error in the patient entry itself, i.e., either the wrong patient is entered or, when entering a patient for the first time, the data is entered incorrectly.”*

(TN 4)

In addition, respondents reported errors due to inaccurate data entry, situations where patients provide false information or cases in which it is not possible to obtain the correct information about the patient’s identity:

*“… that a patient gives you inaccurate information, that’s one thing. It’s another thing that the patient comes in such a state that you can’t get the information that you don’t know who the person is, that you can’t…, basically, I consider it as an administrative error…”*

(TN 6)

Problems also frequently occur when triage nurses do not distinguish between first and last names during triage, especially in non-native patients, which may lead to further errors when entering patient data into the hospital’s information system:

*“Sometimes it’s hard to recognise from the documents when it’s a first name and when it’s a surname. Especially when it comes to foreign patients.”*

(TN 13)

Interviewees described various procedural errors. These include the use of incorrect triage algorithms, the incorrect assignment of patients to inappropriate triage categories, and misjudgment of the severity of the patient’s medical condition, particularly in pain assessment:

*“Definitely, the errors happen, because you can use the wrong algorithm on a patient with the wrong…, wrong triage. You may not put the patient in the right category, the same problem is assessing the pain.”*

(TN6)

Errors also occurred when important information about the main complaints was not obtained from the patient, or when the patient was undertriaged or overtriaged. Other errors included incorrect triage categorisation, inappropriate questioning of patients, lack of patient observation, overlooking symptoms and missing information during patient handover:

*“Let’s say it’s an error, hm, it could be an error to triage too high in the category, or it could be an error not to list the patient’s problems and to downgrade the triage algorithm.”*

(TN11)

Interviewees also highlighted that communication breakdowns between triage nurses and patients, as well as patient misidentification and incorrect questioning, significantly affect triage accuracy:

*“Yes, maybe, I don’t know…, you don’t question the patient properly, you don’t observe him, you miss something.”*

(TN9)

Participants consistently distinguished administrative errors from procedural errors. While administrative errors were primarily associated with patient identification and documentation processes, procedural errors reflected challenges in clinical assessment, prioritisation, and decision-making. Together, these findings suggest that patient safety during triage may be compromised at multiple stages, highlighting the complexity of safe emergency care.

### 3.2. Causes of Errors

The sub-theme of the secondary level, called the cause of the error, comprised three sub-themes of the primary level, namely: (1) Human factor; (2) Organisational factors; and (3) Frequency of patients.

According to the interviewees, errors occur due to human factors such as inattention, fatigue, and reduced concentration, which may lead to incomplete or inaccurate assessments. Although respondents are aware of the potential for errors, they consider the number of errors to be relatively low.

In addition, increasing patient volume and work pressure contribute to stress and fatigue, which negatively affect triage performance. They also point out that triage nurses need to remain focused during busy shifts, as fatigue and stress can negatively impact their ability to listen attentively and accurately assess patients’ health status:

*“Errors will always happen where there is some human factor involved.”*

(TN 14)

*“Yes, I think there are still errors in triage, and there will be errors, mainly because the number of patients is increasing and there are more pressures on triage nurses, …”*

(TN9)

Triage errors can be caused by several factors that affect healthcare professionals’ work environment and work processes. Among these factors, an individual’s experience may lead them to overlook certain details or misjudge a patient’s condition despite their expertise. In addition, situations in which patients are unable to communicate can make accurate assessment difficult, especially when triage nurses rely on information from others, such as paramedics or patient companions. Pressure from multiple directions can lead to a workload that increases stress and may compromise the accuracy and efficiency of the triage process. Distractions during work, such as phone calls or requests for support from colleagues, can also hinder workflow and lead to errors. In addition, pressure from other sources, such as colleagues or paramedics, can further strain the working environment:

*“We also get distracted by phones that interrupt the work process unnecessarily, and by patients who just want to ask a question. Also, colleagues who need help from another colleague. We also get disturbed by paramedics who bring us another patient, of course, they are in a hurry to move on, so they want to hand them over as soon as possible.”*

(TN16)

Errors occur in the triage process, especially at times when there is a large influx of patients. In such circumstances, the constant pressure can cause important details about the patient’s health to be overlooked or neglected. A large influx of patients within a short time frame further increases the pressure on triage, which can lead to errors or the omission of important information. In this context, interviewees point out that it is important to remember that healthcare professionals are only human and may not be aware of all symptoms or situations. Busy days with a high volume of patients can further increase the risk of errors:

*“… these errors most often start to occur after a certain amount of time on the worksite, especially when it’s quite busy, because we want to be fast…”*

(TN 5)

Participants consistently described triage errors as multifactorial. Human factors were closely intertwined with organisational conditions, including workload, interruptions, staffing shortages, and patient volume, indicating that participants perceived errors as emerging from interacting influences rather than a single identifiable cause.

### 3.3. Consequences of Errors

The secondary level sub-theme labelled Consequences of errors is composed of two primary level sub-themes, namely: (1) Patient endangerment and (2) Patient care pathway.

Errors in triage can lead to serious health complications for the patient, which, according to the interviewees, may in turn jeopardise both the patient and the healthcare staff. These errors can pose a significant risk to the patient’s state of health and have irreversible consequences:

*“… you cause him to have some health complications.”*

(TN 3)

*“… then there may be an irreversible situation or irreversible consequences.”*

(TN 8)

Errors in triage can have various consequences that affect the course of patient care in the emergency department. One of these consequences is the prolongation of the patient’s waiting time for appropriate medical care. In addition, a triage error can lead to an incorrect assignment of treatment urgency, which can negatively impact not only the patient who was incorrectly triaged but also another patient who may need immediate treatment. These errors can also affect the course of medical treatment and the time required to examine, diagnose and treat the patient:

*“… his waiting time is extended.”*

(TN5)

*“… affected the progress of the treatment or the timing…”*

(TN8)

Participants perceived the consequences of triage errors as extending beyond individual patients, influencing subsequent clinical management, emergency department workflow, and, in some cases, the safety of healthcare professionals. These findings suggest that the impact of triage errors extends throughout the patient’s emergency care pathway rather than being confined to the initial triage decision.

### 3.4. Accountability Culture

The secondary level sub-theme named Accountability culture is composed of two primary level sub-themes, namely: (1) Accepting or denying the frequency of mistakes and (2) Normalisation of errors as part of daily work.

Interviewers emphasised that errors do occasionally occur; however, they are not entirely sure whether they can claim this is a common phenomenon in patient triage. However, some of the respondents took the opposite view and felt that, unfortunately, errors are quite common. They felt that they face daily challenges that can lead to errors in prioritising patient care. Unfortunately, these errors happen daily and are part of their daily routine:

*“… and some problems simply cannot be prevented.”*

(TN7)

*“It’s certainly likely that an error will happen.”*

(TN17)

Participants described errors as inevitable and a normal part of clinical work, including triage. Although healthcare professionals strive to minimise errors, they perceive them as an inherent aspect of everyday practice. At the same time, participants emphasised the importance of recognising errors, taking appropriate action to reduce their impact, and learning from them. Errors were viewed not only as undesirable events, but also as opportunities for gaining experience and improving professional practice:

*“Of course, there are mistakes, because it’s quite normal that there are errors, hmm.”*

(TN6)

These findings indicate that participants perceived accountability culture as extending beyond individual responsibility. Instead, participants emphasised the importance of recognising errors, discussing them openly, and learning from them within the clinical team, suggesting that accountability was understood as a shared organisational responsibility rather than solely an individual obligation.

### 3.5. Error Prevention

The secondary level sub-theme named Error prevention is composed of four primary level sub-themes, namely: (1) Focus; (2) Professionalism; (3) Consultation & discussion; (4) Staff; and (5) Data checking.

In terms of error prevention, interviewees stated that triage nurses need to be attentive and focussed when entering patient data into the computer system. They must follow algorithms and guidelines closely and be aware of every step they take. They need to be self-critical and realise that even the slightest carelessness can lead to an error. Therefore, triage nurses must constantly focus on their work and endeavour to be as attentive as possible to every detail they enter or write. Increased concentration and constant focus on their tasks can help them avoid mistakes and ensure the accuracy and quality of their work:

*“… we have to be really careful, and then when we write, when we enter data, errors can often be made.”*

(TN3)

Preventing errors in triage was described as requiring a combination of experience, structured training, and ongoing professional development. Participants emphasised that preventing triage errors requires a combination of clinical experience, continuous professional development, and effective communication skills. Mentorship was considered particularly valuable for developing practical competencies during the early stages of triage practice. In addition, effective triage requires strong communication skills, including the ability to ask focused and relevant questions to obtain key information about the patient’s condition. Participants noted that accurate triage depends on both clinical knowledge and the ability to understand the complexity of each case. Overall, preventing errors was seen as a continuous process that extends beyond basic knowledge, requiring ongoing learning, experience, and professional engagement:

*“Definitely experience. That is, enough time in the workplace before the triage even starts.”*

(TN8)

*“… frequent training courses specifically for triage nurses.”*

(TN7)

Participants highlighted the importance of communication and collaboration within the triage team in preventing errors. Regular discussions and exchange of experiences among colleagues were described as key to collective learning and improving triage practices. Consulting with physicians was also considered important, particularly in complex or uncertain cases. Participants emphasised that reflecting on past cases and errors enables identification of underlying causes and supports continuous improvement. Regular team meetings were seen as a valuable mechanism for sharing information, coordinating approaches, and enhancing the overall quality and safety of triage practice:

*“… with discussions, debates among us colleagues, …”*

(TN11)

*“… regular meetings of the triage nurses themselves, not to highlight problems, but to actually talk about what someone has had a case, what someone has noticed, what they have done, what they have done wrong.”*

(TN4)

Effective error prevention in triage relies heavily on adequate staffing support. Additionally, having a sufficiently large team of triage nurses during shifts would allow them to take more breaks, thereby improving concentration and reducing fatigue, both of which are crucial for accurate, safe, and reliable work. This would enable triage nurses to dedicate themselves more fully to their tasks, thereby facilitating better patient care and reducing the risk of errors:

*“In this respect, it would be optimal to have enough of us on triage to be able to give 100% attention to each patient, to have enough of us in the sense of being able to take maybe more breaks during the shift itself…”*

(TN2)

According to the interviewees, ensuring accurate patient identification is paramount in preventing errors in triage. This involves systematically checking documents and identification bracelets at each patient encounter. Participants emphasised that patient identity and clinical information should be verified throughout the triage process, including during patient registration, assessment, and handover, to minimise the risk of misidentification and support continuity of safe patient care.

Overall, participants viewed effective error prevention as requiring a combination of individual competencies and organisational support. Rather than relying on a single intervention, they emphasised that safe triage practice depends on integrating continuous education, teamwork, adequate staffing, sustained attention, and systematic verification procedures.

## 4. Discussion

This study explored triage nurses’ perceptions of patient safety errors during triage, highlighting that these errors are perceived as frequent and multifactorial aspects of triage practice. Rather than reflecting isolated failures in individual performance, participants described patient safety errors as arising from the interaction of individual clinical judgement, organisational conditions, and contextual factors such as workload and patient volume. Unlike previous studies, which have primarily focused on individual aspects of patient safety in the emergency department—such as triage accuracy, workload, communication failures, interruptions, patient identification errors, and other factors associated with triage errors [2,4,10,11,12,13], this study provides qualitative insight into how triage nurses themselves conceptualise patient safety errors and perceive the interactions among individual, organisational, and contextual factors that contribute to these errors. These findings suggest the complexity of safe decision-making in emergency care and may support the need for system-level approaches to patient safety. Participants identified several strategies to mitigate errors, including adequate staffing, continuous training, interprofessional communication, and systematic patient identification. Together, these findings suggest that participants perceived patient safety in triage as a shared organisational responsibility rather than solely an individual clinical responsibility.

Patient safety is a fundamental prerequisite for quality healthcare, as it prevents errors in healthcare delivery and protects patients from potential harm [23,24]. Our findings indicate that triage errors are perceived as an inherent part of clinical practice, although their frequency varies according to individual experiences. Nevertheless, some interviewees reported that the number of errors is relatively low while acknowledging that they still occur occasionally. Triage error rates vary widely in the literature, and the differences in triage systems and definitions across institutions make comparisons challenging [10]. Different studies have found that errors in the triage system occur at different percentages, with errors identified in 11% [25], 14% [6], 16% [2] and even 49% [26] of the analysed cases. Importantly, participants associate errors with prolonged waiting times and potential patient harm, underscoring the critical role of triage in ensuring safe and timely care. Ausserhofer et al. [2] found that errors in triage were not associated with patient mortality, but patients whose triage included errors spent more time in the emergency department and were hospitalised more often than patients whose triage did not include errors. This finding further supports a systems perspective on triage safety by demonstrating that patient safety errors emerge from interactions among individual, organisational, and contextual factors.

Beyond the perceived frequency of errors, participants also described different types of patient safety errors occurring during triage. Our findings highlight that administrative and procedural errors represent two complementary dimensions of patient safety during triage. Administrative errors were primarily related to patient misidentification and inaccurate data entry. Similarly, Riplinger, Piera-Jiménez and Dooling [9] identified patient identification errors as one of the most common adverse events in nursing practice, particularly during patient admission and documentation processes. These errors often arise in high-pressure environments characterised by high patient turnover and complex workflows. Procedural errors primarily reflected challenges in clinical assessment and decision-making under conditions of uncertainty. Consistent with previous research, procedural errors included both undertriage and overtriage, each of which may adversely affect patient outcomes and resource utilisation [10,27]. Furthermore, the study by Wolf et al. [28] found that nurses in the emergency department often triage the ED rather than the patient, thereby manipulating the triage scoring system according to the situation in their emergency department. Triage in the emergency department requires interdisciplinary collaboration between healthcare providers who may have differing views (e.g., physicians may feel that nurses did not triage appropriately, while nurses may feel that physicians did not respond promptly to high-acuity patients) [4]. Therefore, in triage systems, triage nurses play a crucial role in correctly assessing the severity of the patient’s condition and limiting errors such as over- or undertriage, which can lead to poor patient outcomes [2]. Similar procedural errors, including incorrect algorithm selection and inappropriate triage categorisation, have been consistently reported in previous studies [2,8]. Previous studies have consistently shown that limited clinical experience, stress, insufficient knowledge, and inadequate training increase the likelihood of triage errors [29,30,31]. Consistent with these findings, our participants also emphasised that inexperienced staff and nursing shortages increase the risk of errors, particularly when triage responsibilities are assigned to healthcare professionals without sufficient triage expertise [32].

According to Soontorn, Sitthimongkol, Thosingha and Viwatwongkasem [31], the level of professional experience among triage nurses in the emergency department is associated with errors in triage category assignment, i.e., triage nurses with less than five years of professional experience are more likely to make errors. Staff shortages in nursing can lead to triage tasks being assigned to healthcare professionals who lack the knowledge, experience, and training required for triage [32]. Together, these findings suggest that procedural errors are influenced not only by individual clinical competence but also by organisational support for ongoing education and decision-making, reinforcing the importance of continuous professional development in triage practice. In Slovenia, triage nurses receive initial training [19]; however, ongoing training is not consistently required, which may represent a gap in maintaining competence. Peta, Day, Lugari, Gorman, Ahayalimudin and Pajo [5] emphasise that continuous training is essential to support appropriate clinical decision-making.

Rather than viewing triage errors as isolated mistakes made by individual nurses, participants consistently described them as emerging from dynamic interactions between cognitive, organisational, and environmental factors. Participants consistently associated triage errors with demanding working conditions characterised by time pressure, cognitive overload, interruptions, and high patient volume. These organisational pressures were perceived to reduce nurses’ capacity to perform accurate triage assessments, thereby increasing the likelihood of patient safety errors. In addition, Pryce et al. [33] note that patients queuing for triage represent a bottleneck that can jeopardise patient care. Ausserhofer et al. [2] highlight similar factors related to the patient, work environment, and healthcare professionals. The most common causes of healthcare errors include fatigue [34], distractions or interruptions in the work process [35], workload and working hours [30,36], and inadequate staffing and overloading nurses with non-triage related duties [34]. Our findings extend these observations by suggesting that triage nurses perceived errors as arising not only from individual performance but also from the interaction of multiple organisational and environmental factors. From an interpretative perspective, this understanding appears consistent with the Human Factors Framework, which recognises that clinical performance is influenced by interactions between individual capabilities, task demands, environmental conditions, teamwork, and organisational systems [37]. These findings may suggest that improving triage safety may require redesigning work systems and organisational processes rather than relying solely on individual vigilance. Participants described how cognitive overload, frequent interruptions, competing demands, and heavy workloads reduced their ability to perform accurate triage assessments, highlighting the complex relationship between individual decision-making and the surrounding work environment.

To further interpret participants’ accounts, we considered Reason’s Swiss cheese model of patient safety, which conceptualises adverse events as the consequence of multiple interacting latent and active failures rather than isolated individual mistakes [38]. These findings suggest that staffing shortages, communication challenges, interruptions, and organisational pressures interact to create conditions in which individual triage errors become more likely. This interpretation suggests that preventing triage errors may require strengthening multiple organisational safety barriers rather than relying on individual healthcare professionals to compensate for system weaknesses. From a theoretical perspective, our findings may also be interpreted in light of the principles of High Reliability Organisations, which emphasise continuous learning, collective mindfulness, resilience, and proactive identification of system vulnerabilities [39]. These empirical findings can also be interpreted through patient safety culture frameworks that emphasise teamwork, psychological safety, organisational learning, open communication, and shared responsibility for patient safety [40]. Participants’ emphasis on teamwork, ongoing education, organisational support, and learning from errors rather than assigning individual blame reflects key characteristics of resilient healthcare organisations committed to maintaining patient safety under complex and high-risk conditions [41].

Building on these findings, participants identified several organisational strategies that they perceived may contribute to reducing the occurrence of triage errors. Errors that may occur during the triage process affect the patient’s overall pathway through the emergency department and may also impact their entire hospital stay [8]. According to other authors, triage errors might increase mortality risk by up to 18% [8,42]. These findings suggest that establishing error prevention and continuous improvement procedures may support safer triage practice. Participants highlighted organisational strategies, including adequate staffing, continuous professional development, interprofessional communication, and systematic patient identification, as approaches that may contribute to safer triage practice. Similarly, previous studies suggest interventions such as training programmes, audit and feedback, and decision-support systems [4,43]. According to Bérubé [4], these approaches can affect nurses’ ability, motivation, and opportunities to implement best practices in triage. This system-oriented perspective is also consistent with recent international recommendations advocating structured system-level approaches to patient safety in emergency departments. In particular, the recently developed Emergency Department Safety Checklist emphasises standardised patient identification procedures, structured communication during clinical handovers, regular reassessment, medication safety, and systematic review of safety-critical processes throughout the patient’s journey in the emergency department [44]. In combination with structured feedback processes and continuous quality improvement activities [4], these structured interventions may strengthen organisational learning, promote consistent triage practice, and further reduce the likelihood of patient safety errors during triage.

An important finding of this study was that many participants perceived errors as an inevitable part of everyday triage practice. While this perception may reflect the realities of working in a high-pressure emergency environment, it also suggests that repeated exposure to demanding clinical conditions may influence how healthcare professionals perceive and respond to errors in everyday clinical practice. From an interpretative patient safety perspective, this finding highlights the importance of fostering a just and non-punitive safety culture that encourages open discussion, reporting, and organisational learning rather than attributing blame solely to individual healthcare professionals.

Consistent with this interpretation, triage health professionals play an important role in the occurrence of triage errors but are not solely responsible for them. According to Aghabarary et al. [45], there is often a tendency to attribute blame to individuals when errors occur. However, recognising errors as learning opportunities may promote reporting and improvement [46]. Therefore, Karaca et al. [47] emphasise that errors should be reported, while responsibility also lies with organisational systems. Healthcare institutions should promote a culture of safety, including confidentiality, root cause analysis, and shared learning. Karaca, Akin and Harmanci Seren [47] highlight that all healthcare professionals working in the institution, including triage healthcare professionals, should be encouraged to work in harmony, maintain constant communication and promote mutual reporting of errors. Moreover, reporting errors and problems should be a commitment of everyone working in the institution.

While previous qualitative studies have identified a range of factors contributing to triage errors, including workload, interruptions, communication challenges, and clinical decision-making [4,5,28], our findings suggest that these factors should not be interpreted as independent sources of error. Instead, participants consistently described them as dynamically interacting within the organisational context of emergency care. This system perspective extends previous qualitative evidence by demonstrating how triage nurses understand patient safety as a product of interconnected organisational, contextual, and individual influences.

Our findings may have important implications for clinical practice, organisational management, and healthcare policy. The findings suggest that strengthening patient safety during triage may benefit from organisational investment in adequate staffing, protected time for continuing triage education, regular simulation-based training using realistic triage scenarios, structured mentoring of newly appointed triage nurses, and regular multidisciplinary case reviews that support organisational learning. At the policy level, healthcare systems may consider establishing standardised requirements for ongoing triage competency assessment, regular refresher training, and structured reviews of triage-related patient safety incidents to promote consistent triage practice across emergency departments. In addition, healthcare organisations may benefit from promoting a Just Culture that encourages open reporting and discussion of triage errors while using these events to improve organisational processes rather than attributing blame to individual healthcare professionals. Such a systems-oriented approach may contribute to more sustainable improvements in triage quality, patient safety, and organisational learning. These organisational and policy measures may help reduce variation in triage practice, strengthen patient safety, and support consistent quality of emergency care across healthcare settings. Although these findings were generated within a single Slovenian university hospital using the Manchester Triage System, many of the organisational challenges identified in this study, including workload, staffing, communication, and patient identification, are common across emergency departments internationally. Nevertheless, differences in organisational culture, staffing models, available resources, and national emergency care systems may influence how these findings are transferred to other healthcare settings. Consequently, patient safety interventions should be adapted to local organisational contexts while maintaining the systems-based principles identified in this study.

### Limitations

Although the findings provide valuable insights into triage nurses’ perceptions of patient safety errors, several limitations should be considered when interpreting the results. The recruitment process was restricted to triage nurses working exclusively within a single ED, which may limit the transferability of the findings to other settings. To mitigate this limitation, purposive sampling was used to recruit participants with diverse ages, years of emergency nursing experience, and triage experience, thereby capturing a broad range of perspectives within the study setting. Nevertheless, future multi-site qualitative studies involving different emergency departments and healthcare systems are recommended to enhance the transferability of the findings. The findings should therefore be interpreted within the context of a tertiary emergency department in a high-income European healthcare system. Organisational structures, staffing levels, available resources, and triage procedures may differ substantially in lower-resource healthcare settings or in hospitals operating within different healthcare systems, potentially affecting the transferability of these findings.

Additionally, participants’ individual experiences and backgrounds may have influenced how they expressed and interpreted their perspectives. The data provided by the participants reached saturation, meaning that no new information or perspectives were emerging. However, the perspectives of non-participants may differ, potentially limiting the range of views captured. All participants worked within the Slovenian healthcare system using the Manchester Triage System (MTS). While this ensured methodological consistency, it may limit the transferability of the findings to healthcare systems using different triage models, organisational structures, or national emergency care policies. Future multi-site studies, including diverse healthcare systems and triage models, are warranted to further the transferability of these findings across different organisational contexts. The study relied solely on interview data without methodological triangulation, which may limit the depth and validation of the findings. Consequently, the findings reflect participants’ self-reported perceptions and experiences rather than directly observed or objectively measured triage errors. As a result, the study provides insight into how triage nurses perceive patient safety errors rather than the actual frequency or occurrence of such errors in clinical practice.

In addition, interviews were conducted in the workplace environment, which may have influenced participants’ responses and led to socially desirable answers. Participants may therefore have underreported errors or expressed views perceived as professionally acceptable despite assurances of confidentiality. The interviewer’s clinical background in emergency care may have influenced both data collection and interpretation, although reflexive awareness was maintained throughout the research process. A semi-structured interview guide was used to promote consistency during data collection; however, some degree of interviewer influence cannot be completely excluded. Furthermore, transcripts were not returned to participants for verification, which may affect the credibility of the findings. These methodological limitations should be considered when interpreting the findings, as they may have influenced both the content and depth of participants’ responses. Nevertheless, the consistency of themes across interviews and the achievement of data saturation support the credibility of the findings.

The study did not include patients as stakeholders in the triage process. Incorporating patients’ perspectives could provide additional insights into the experience of care and perceptions of safety during triage. Despite these limitations, the findings provide important insights into triage nurses’ perceptions of patient safety errors and offer practical implications for improving patient safety in emergency departments. Future research should also consider methodological triangulation by combining interviews with observations, document analysis, or additional stakeholder perspectives, including patients and other healthcare professionals, to gain a more comprehensive understanding of triage safety.

## 5. Conclusions 

Triage is a complex and safety-critical process in which patient safety errors may occur at multiple stages of care. This study identified two broad categories of patient safety errors during triage—administrative and procedural—and demonstrated that triage nurses perceived these errors as arising from interacting human, organisational, and system-level factors rather than isolated individual failures. Participants emphasised that improving patient safety may require not only individual clinical competence but also organisational support through adequate staffing, continuous professional development, effective communication, and a non-punitive culture that encourages learning from errors. These findings reinforce the importance of adopting systems-based approaches to patient safety in emergency care and provide practical insights for strengthening the quality and safety of triage practice. Future research should explore these findings across different healthcare settings and triage systems and examine the effectiveness of organisational interventions designed to reduce triage-related patient safety errors.

## Figures and Tables

**Table 1:** Overview of themes and subthemes identified during thematic analysis.

Main Theme	Second-Level Subtheme	Primary Level Subtheme	Representative Codes
Perceptions of patient safety errors during triage encounter	Types of errors	Administrative	Patient misidentification; registration and documentation errors; inaccurate patient data.
		Procedural	Incorrect triage categorisation; inappropriate algorithm selection; incomplete assessment; under-/overtriage.
	Causes of errors	Human factor	Fatigue; reduced concentration; clinical inexperience; communication barriers.
		Organisational factors	High workload; organisational pressure; workflow interruptions.
		Frequency of patients	High patient volume; overcrowding; waiting patients.
	Consequences of errors	Patient endangerment	Patient health deterioration; patient harm; risk to healthcare staff.
		Patient care pathway	Delayed patient care, incorrect patient prioritisation, and disrupted treatment process.
	Accountability culture	Accepting or denying the frequency of mistakes	Perceived frequency, uncertainty, and inevitability of errors.
		Normalisation of errors as part of daily work	Errors as a normal part of practice; acknowledgement and perceived inevitability of errors.
	Error prevention	Focus	Sustained attention; algorithm adherence; self-awareness.
		Professionalism	Clinical experience; continuous professional education; communication competencies; mentorship.
		Consultation, discussion	Interprofessional communication; collaborative discussion; consultation with physicians.
		Staff	Adequate staffing/staff shortages; staffing levels.
		Data checking	Patient identity and information verification; data verification.

## Data Availability

Additional data from this study are not publicly available in order to maintain participants’ anonymity but can be provided on request.

## Supplementary Materials

The following supporting information can be downloaded at https://www.mdpi.com/article/10.3390/healthcare14152291/s1, Figure S1: Coding tree with representative codes for each subtheme; Table S1: Examples of the analytical process from meaning units to themes.
