# Supplementary material for: Triage Nurses’ Perceptions of Patient Safety Errors During Triage in the Emergency Department: Qualitative Thematic Analysis Study
Source: Healthcare (Basel). 2026 Jul 29;14(15):2291. doi: 10.3390/healthcare14152291 (PMC13465030; doi:10.3390/healthcare14152291)
Supplement: Supplementary file 1 [file healthcare-14-02291-s001.zip › healthcare-4405181-supplementary.pdf]

**Figure S1.** Coding tree with representative codes for each subtheme.

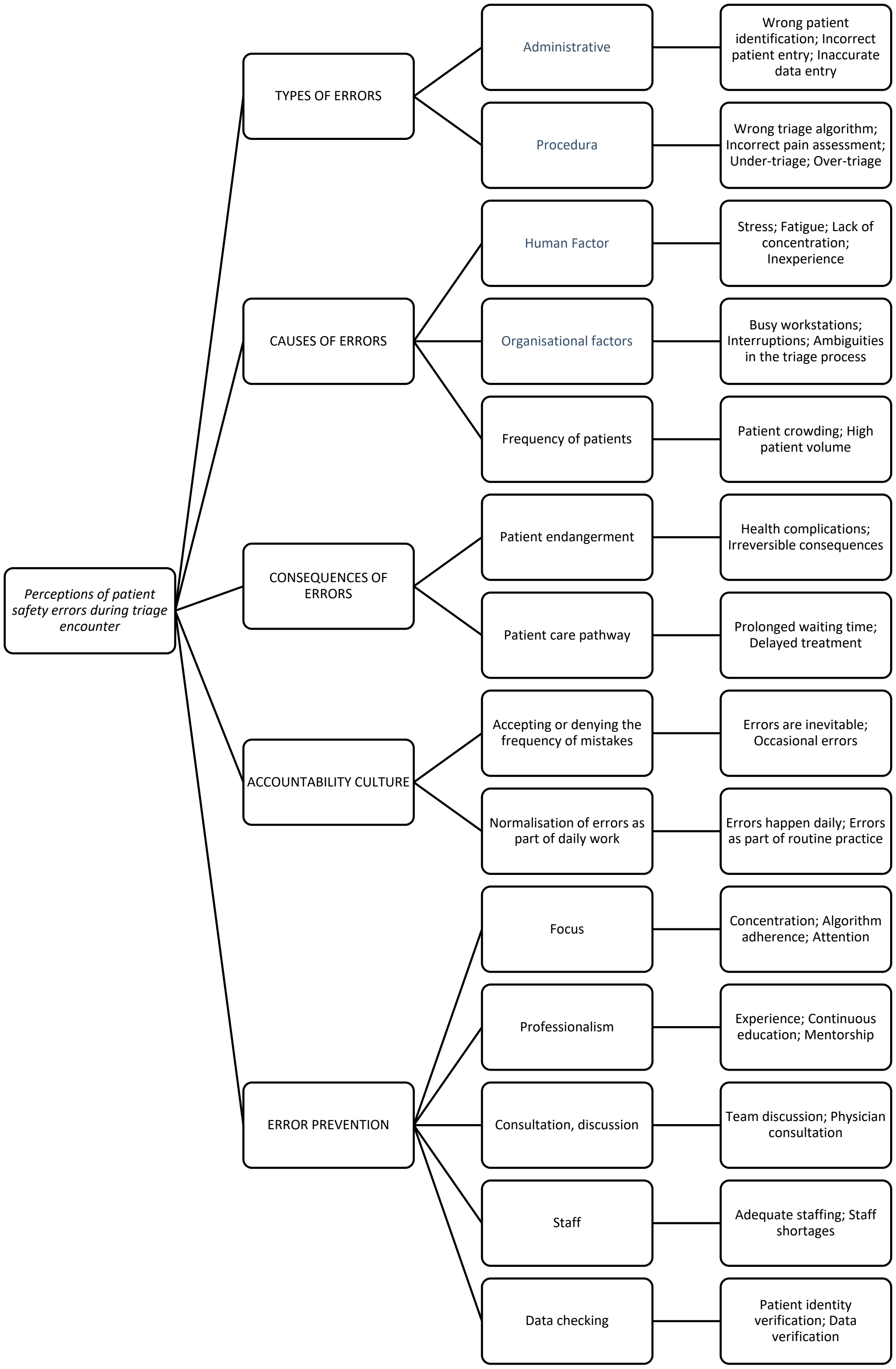

**Table S1.** Examples of the analytical process from meaning units to themes.

| **Meaning unit** | **Initial code** | **Primary – level subtheme** | **Second level subtheme** | **Main theme** |
|---|---|---|---|---|
| "...either the wrong patient is entered or the data are entered incorrectly." (TN4) | Wrong patient identification; incorrect patient entry | Administrative | Types of errors | Perceptions of patient safety errors during triage encounter |
| "You can use the wrong algorithm… or not put the patient in the right category." (TN6) | Wrong triage algorithm; incorrect categorisation | Procedural | Types of errors | Perceptions of patient safety errors during triage encounter |
| "Errors will always happen where there is some human factor involved." (TN14) | Human error; stress | Human factors | Causes of errors | Perceptions of patient safety errors during triage encounter |
| "Phones interrupt the work process… colleagues and paramedics also interrupt us." (TN16) | Interruptions; workplace distractions | Organisational factors | Causes of errors | Perceptions of patient safety errors during triage encounter |
| "...when it's quite busy, because we want to be fast..." (TN5) | High patient volume; workload pressure | Frequency of patients | Causes of errors | Perceptions of patient safety errors during triage encounter |
| "...his waiting time is extended." (TN5) | Delayed care | Patient care pathway | Consequences of errors | Perceptions of patient safety errors during triage encounter |
| "...there may be irreversible consequences." (TN8) | Patient harm | Patient endangerment | Consequences of errors | Perceptions of patient safety errors during triage encounter |
| "Of course there are mistakes… it is quite normal." (TN6) | Errors are inevitable | Normalisation of errors as part of daily work | Accountability culture | Perceptions of patient safety errors during triage encounter |
| "...frequent training courses specifically for triage nurses." (TN7) | Continuous education | Professionalism | Error prevention | Perceptions of patient safety errors during triage encounter |
| "...we have to be really careful when we enter data." (TN3) | Patient identity verification; careful data checking | Data checking | Error prevention | Perceptions of patient safety errors during triage encounter |
